# Do full leg compression sleeves improve repeated sprint performance after soccer‐specific exercise in adolescent male soccer players?

**DOI:** 10.14814/phy2.70778

**Published:** 2026-02-15

**Authors:** Florian A. Engel, Claudia Kubica, Stefan Altmann, Rainer Neumann, Billy Sperlich

**Affiliations:** ^1^ Integrative and Experimental Exercise Science and Training University of Wuerzburg Würzburg Germany; ^2^ Institute of Sport Science University of Bern Bern Switzerland; ^3^ Institute of Sports and Sports Science Karlsruhe Institute of Technology Karlsruhe Germany; ^4^ TSG ResearchLab gGmbH Zuzenhausen Germany; ^5^ Institute of Movement and Sport Karlsruhe University of Education Karlsruhe Germany

**Keywords:** athletes, compression garments, DOMS, muscle soreness, repeated sprinting

## Abstract

This study examines the effects of full‐leg compression sleeves worn during a 90‐min recovery period on repeated sprint performance and exercise‐induced leg soreness (DOMS) in youth soccer players. Twelve male youth soccer players (17 ± 0 years; 178 ± 7 cm; 70.9 ± 7.5 kg) performed a repeated sprint protocol (5 × 30 m sprints, 20 s recovery; RSP_1_) followed by a modified Loughborough Intermittent Shuttle Test (LIST) to induce fatigue. After the LIST, players underwent a 90‐min passive recovery wearing either full leg compression sleeves (COMP, 19–25 mmHg) or regular gym pants (CON) in a randomized crossover design. After the 90‐min recovery, all players repeated the RSP (RSP_2_), and exercise‐induced DOMS was assessed via a visual analogue scale 14 and 24 h post‐exercise. Mean sprint times were similar across conditions (RSP_1_: COMP 4.59 ± 0.16 s, CON 4.65 ± 0.18 s; RSP_2_: COMP 4.59 ± 0.15 s, CON 4.64 ± 0.19 s), with no significant differences between COMP and CON for performance changes (COMP: +0.01 ± 0.06 s; CON: −0.01 ± 0.05 s) or DOMS (14 h: COMP 3.49 ± 1.73, CON 4.73 ± 2.32; 24 h: COMP 2.78 ± 2.32, CON 4.04 ± 2.12). Compression garments had no impact on repeated sprint performance or exercise‐induced leg soreness. The efficacy of compression garments for recovery remains inconclusive, requiring further research.

## 1 INTRODUCTION

Compression garments are typically employed to improve hemodynamics (MacRae et al., 2011; Valle et al., 2013) and reduce edema (Valle et al., 2013), are easy to apply at low costs and are gaining popularity among athletes to improve various dimensions of performance and recovery (Born et al., 2013; Engel et al., 2016; MacRae et al., 2011). Numerous studies have investigated the application, utility, as well as performance (Born et al., 2013; Engel et al., 2016) and recovery effects (Brophy‐Williams et al., 2017; Brown et al., 2017; Hill et al., 2014; Sperlich et al., 2013) induced by various forms of compression attire. According to the current evidence, the application of compression garments seems to moderately improve post‐exercise recovery, most effective for long‐term recovery (>24 h) following exercise‐induced muscle damage (Brown et al., 2017; Hill et al., 2014). These results are supported by other studies, demonstrating the positive effect of compression garments applied during exercise (Ali et al., 2007; Born et al., 2014; Valle et al., 2013), or 24 h after exercise on delayed onset of muscle soreness (DOMS) (Ali et al., 2007; Goto et al., 2017; Maruyama et al., 2019), or on enhanced recovery of neuromuscular performance (Hill et al., 2017; Mizuno et al., 2016).

Even though soccer is very popular, only a few studies investigated the compression‐mediated recovery of trained soccer players from soccer‐specific training and/or recovery from (simulated) match play (Marqués‐Jiménez, Calleja‐González, Arratibel, et al., 2018; Marqués‐Jiménez, Calleja‐González, Arratibel‐Imaz, et al., 2018; Otten et al., 2019). In soccer, it is essential to maximize match performance and maintain repetitive high‐intensity short‐duration efforts (Bradley et al., 2009) (19, 20), including short sprints, jumps, and multidirectional movements (Carling, 2010; Harper et al., 2019; Scott et al., 2014; Taylor et al., 2017) as the key basis for high‐quality shots, turns, one‐on‐ones, balance, and ball handling.

After a regular match and depending on the biological sub‐system (e.g. humoral, hormonal, neuro‐muscular, metabolic etc.), soccer players usually reach full recovery after 72 h (Silva et al., 2018). Additionally, in modern‐day soccer, a dense schedule of training sessions and matches with high internal and external loads are common for players (Barnes et al., 2014; Bush et al., 2015; Carling et al., 2015; Julian et al., 2021). The overall load may result in incomplete recovery and reduced match performance (Nunome et al., 2013), and inadequate time for rest and recovery between matches and/or training may expose players to a higher risk of injury (Christopher Carling et al., 2018; Nédélec et al., 2013).

Scientific studies analyzing post‐exercise recovery methods in youth soccer players are relatively sparse. A recent systematic review and meta‐analysis (Calleja‐González et al., 2021) reported no significant effects of water immersion protocols on the recovery of neuromuscular performance in this population. However, these protocols, including cold water immersion, positively impacted exercise‐induced muscle damage, inflammatory responses, and perceptual parameters. Notably, the review did not include studies analyzing the use of compression garments during the post‐exercise recovery period.

Compression garments (compression tight combined with full leg compression sleeves, 31.4 ± 11.1 mmHG (33); compression socks, ~19–23 mmHG (Brophy‐Williams et al., 2017)) worn during a 60 min recovery period between two bouts of endurance exercise appear to be beneficial for performance output in the second bout (Brophy‐Williams et al., 2017; Lee et al., 2021). The application of compression socks during a 60‐min recovery period between two time trials of 5 km running (Brophy‐Williams et al., 2017) or after a 20 min fatiguing cycling protocol (Lee et al., 2021) considerably diminished the performance decrease after the 60‐min recovery period. Although it seems as if the belief of participants a priori in the effectiveness of compression clothing for performance enhancement can unleash a considerable potential (Brophy‐Williams et al., 2017). Repeated sprint performance following exhausting repeated sprint exercises and a recovery period of 48 h wearing compression tights with 10 and 25 mmHg had no effect on performance for handball players (Zinner et al., 2017).

The positive effects of compression garments on post‐exercise recovery may be attributed to biological, physiological, psychological and biomechanical mechanisms (Born et al., 2013; Brophy‐Williams et al., 2017, 2019; Engel et al., 2016; Lee et al., 2021; Valle et al., 2013). The application of compression garments increases venous return (Bottaro et al., 2011; Ibegbuna et al., 2003), stroke volume and cardiac output (Lee et al., 2021), causing an enhanced supply of muscles with oxygen and nutrients, as well as augmented elimination of waste products and metabolites (Bottaro et al., 2011; Lee et al., 2021). Conversely, a review found only small effects in the elimination of blood lactate levels wearing compression garments during or following running but reduced perceived exertion levels during running and lower levels of post‐running DOMS (Engel et al., 2016). Lower levels of perceived exertion during exercise and lower levels of post‐exercise DOMS were found in numerous studies (Brown et al., 2017; Hill et al., 2014, 2017; Lee et al., 2021). A study showed reduced muscle displacement, reduced soft tissue vibrations, and lower muscle activation in the lower limbs while running with long compression tights compared to running without compression clothes (Broatch et al., 2020). Together with increased blood flow velocity and improved lymphatic circulation (Bottaro et al., 2011), these mechanisms may contribute to lower levels of perceived exertion and lower levels of post‐exercise DOMS. Additionally, reduced inflammation (Ali et al., 2007; Hill et al., 2014) and reduced pain perception (Engel et al., 2016; Kraemer et al., 2001) are attributed to limited and less available space for swellings created by the external pressure gradient of compression clothing.

While the effects of compression garments on endurance performance were analyzed extensively, the effects of compression garments on repeated sprint performance remain unclear. This is particularly relevant for highly trained youth soccer players who frequently engage in repetitive high‐intensity efforts of short duration with limited recovery periods, such as repeated sprinting, during competitive matches (Buchheit et al., 2010). No positive effects of compression garments were observed on repeated sprint performance following an exhausting repeated sprint exercise and a 48‐h recovery period, regardless of whether compression garments were worn, in well‐trained handball players (Zinner et al., 2017). These findings are supported by Goto et al. (2017), who reported that the use of a whole‐body compression suit between two sessions of exhaustive repeated sprinting, jumping, and resistance exercise, separated by 4 h of rest, did not influence repeated sprint performance (Goto et al., 2017). Similarly, in a study examining the effects of compression garments worn between two training sessions, athletes participated in a simulated intensive 80‐min team sport training session and subsequently wore compression clothing for 24 h. The results indicated no significant impact of compression garments on repeated sprint performance, assessed 24 h after the simulated training session, compared to the control condition (Duffield et al., 2008).

Although the body of research regarding compression clothing is increasing, most research has focused on adult (elite) athletes (Brown et al., 2017; Hill et al., 2014; Marqués‐Jiménez et al., 2016; Nédélec et al., 2013). Furthermore, most of the compression‐related research investigated the direct performance enhancement of compression garments during exercise but data about the effects of compression garments worn between two exercise sessions with short recovery periods (60–90 min) are still sparse (Brophy‐Williams et al., 2017; Lee et al., 2021).

Therefore, the present study aimed to assess the effects of wearing full leg compression sleeves during a 90‐min recovery period following a soccer‐specific fatiguing exercise on performance in a subsequent repeated sprint protocol and postexercise DOMS in youth soccer players.

We hypothesize that the application of full leg compression sleeves during recovery after a soccer‐specific fatiguing exercise will enhance recovery and thereby reduce the performance change in the repeated sprint protocol following the soccer‐specific fatiguing exercise and that the post‐exercise DOMS will be lower.

## 2 MATERIALS AND METHODS

### 2.1 Participants

A total of 12 well‐trained male youth soccer players, classified as tier 2 athletes (McKay et al., 2022) (mean ± SD; age: 17 ± 0 years; body height: 178.3 ± 6.9 cm, body mass: 70.9 ± 7.5 kg), volunteered to participate in the present study. All participants were accustomed to four training sessions (90–120 min duration each with varying low‐ to high‐intensity) and one match (90 min) per week. All players competed at the regional level and did not present any medical conditions or acute or chronic injuries during the investigation. All participants and their legal guardians provided written consent to participate after being informed of the benefits and risks involved and were free to withdraw from the study at any time with no further consequences. The study was approved by the ethical review board of the master program Exercise Science and Training, Julius‐Maximilians‐Universität Würzburg, Germany. This study was conducted in accordance with the principles of the Declaration of Helsinki (“World Medical Association Declaration of Helsinki: Ethical Principles for Medical Research Involving Human Subjects,” 2013).

### 2.2 Experimental protocol

**FIGURE 1:**
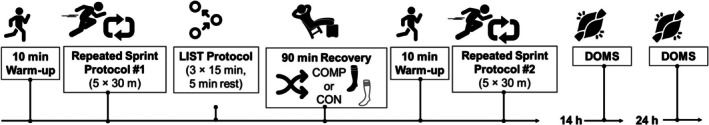
Experimental protocol of the counter‐balanced, controlled crossover study. COMP, compression condition (wearing full leg compression sleeves during recovery period); CON, control condition (wearing no compression sleeves during recovery period); DOMS, delayed onset of muscle soreness; LIST Protocol, modified Loughborough Intermittent Shuttle Test.

The CONSORT checklist is provided as a Supplementary File (Schulz et al., 2010). The experimental protocol comprised a counter‐balanced, controlled crossover design. Within 2 weeks the players reported twice to the exercise laboratory (ambient conditions: 20°C–22°C and 40%–50% relative humidity). The players were randomly assigned into two groups in a crossover design. The random allocation sequence was generated by artificial intelligence. The same procedures were performed during the two experimental sessions and separated by 6 days. Based on the crossover design half of the group either wore full‐leg compression sleeves (compression condition) or wore their long‐sleeve gym pants exerting no compression onto the leg muscles (control condition) during the 90‐min recovery period (see Figure 1).

For practical reasons, the players were divided into three groups, each started the experiments at different times. The order of the experimental conditions was randomized between the three groups, meaning that all members of a given group performed the same condition in the same order. The orders and start times of each group and participants were kept identical for the two experimental sessions. Each experimental session consisted of a standardized warm‐up, followed by an initial repeated sprint protocol (RSP_1_). After the RSP_1_, a modified Loughborough Intermittent Shuttle Test (LIST) lasting 45 min was performed to induce an intense soccer‐specific load. Following the LIST protocol, soccer players had 90 min of passive recovery (see Figure 1). During the recovery period, participants wore either compression sleeves (compression condition) or conventional gymnastic pants (control condition) without any additional compression. After the recovery period, the identical repeated sprint protocol (RSP_2_) was repeated to assess the potential impact of compression sleeves worn during recovery on subsequent repeated sprint performance. The warm‐up, RSP, and LIST procedures were incorporated into the training routine 1 week before the first experimental session to warrant habituation, aiming to minimize potential errors. To limit the impact of diurnal variations and to warrant sufficient recovery between sessions, the habituation session as well as the two experimental sessions were performed in the evening during the regular training hours (5:00–7:00 p.m.) of the soccer players, with 6 days between each session. The coach of the soccer players was instructed to keep all training identical for 48 h before testing on all occasions. Participants were additionally asked to refrain from strenuous exercise (<24 h) and to arrive in a fully rested, hydrated state. All testing procedures were performed indoors at the same facility and participants wore the same footwear and exercise attire for every session.

### 2.3 Warm‐up

As preparation for RSP_1_ and RSP_2_, participants performed a standardized 10 min warm‐up protocol, as instructed by the investigators. The warm‐up protocol consisted of slow jogging, running drills, three short accelerations, three short sprints, lunges, mobility drills, and one 30‐m sprint.

### 2.4 Repeated‐Sprint‐protocol

The RSP is a standardized test reflecting the intermittent nature of team sports to evaluate the repeated sprint ability (Altmann et al., 2018; Altmann et al., 2019). RSP consisted of 5 × 30 m maximal sprints interspersed by 20‐s active recovery between sprints. Before RSP_1_ and RSP_2_, all players were instructed to perform each sprint with maximal effort over the given distance and to avoid a finishing dip or an early deceleration. The starting position was a split start and rocking movements or leaning back prior to sprinting were not allowed. Considering the 20‐s recovery interval between the sprints during both RSPs, all players were instructed to decelerate after completing each 30‐m distance and jogging back to the start. During the last 10‐s of each 20‐s rest interval, a loud countdown was provided by the investigators. All players started each 30‐m distance from a standing position, thereby avoiding a flying start. During RSP_1_ and RSP_2_, standardized verbal encouragement was provided by the investigators following each of the five sprints. Both the RSP and the habituation session were performed indoors on the same PVC floor. All 5 × 30‐m sprint times were recorded with validated single‐beam timing lights, employing error correction–processing algorithms sampling at 1.000 Hz (Smartspeed Pro, Fusion Sport, Coopers Plains, Australia) (Altmann et al., 2018). The timing lights were placed at 0, 5, 10, and 30 m mounted at 95 cm height, representing the height of the body close to the center of mass (Haugen & Buchheit, 2016). The starting distance from the first timing gate was set at 30 cm (Altmann et al., 2015).

### 2.5 Loughborough intermittent shuttle test

A modified version of the LIST was performed (Nicholas et al., 2000) to simulate the activity patterns of a soccer training session and to induce a demanding soccer‐specific load (see Table 1). The procedure included 3 × 15 min of running with varying intensities, interspersed by 3 min rest periods after each 15 min. During the 15‐min periods, the participants were required to perform running intervals at various intensities between two lines, 20 m apart, at an indoor track. The durations and intensities of the 15‐min running periods were standardized using the BORG Scale (6–20) according to the following repetitive scheme:

**TABLE 1:** Protocol of the modified Loughborough Intermittent Shuttle Test.

Number/Distance or duration	Exercise mode	Intensity on Borg scale (6–20)
3 × 20 m	Walking	7
1 × 20 m	Sprinting	20
4 s	Passive recovery	6
3 × 20 m	Easy running	12
3 × 20 m	Intensive running	16
3 min	Passive rest	6

Abbreviation: BORG, rates of perceived exertion on the 6–20 Borg scale (Borg, 1982).

Two large flipcharts with the schematic overview of the LIST protocol (Table 1) were placed beside the 20‐m track to assist the players in memorizing the LIST protocol. The players had to repeat this protocol for 15 min and then rest for 3 min. Each player performed overall three of the 15 min cycles. The investigators monitored the duration of each 15 min cycle and the 3 min recovery periods with a stopwatch (PC‐90, schütt‐sport©, Marburg, Germany) and provided verbal information about the remaining duration to each player. To document internal load during the LIST protocol, a subsample of five participants wore heart rate monitors and chest strap (Polar H7, Polar Electro© Oy, Kempele, Finland) during the LIST protocol to record heart rates continuously.

### 2.6 Compression garments

All players completed one recovery period wearing new compression leg sleeves, the product *Total Full Leg* (68% Polyamide, 23% Elasthan, 9% Polystyrol) from the manufacturer Compresssport (Nyon, Switzerland). To ensure adequate fitting of the compression sleeve, calf and thigh circumferences from each participant were assessed with a measuring tape at the spot of the largest circumference. Maximal circumferences of the calf and the thigh were measured on the dominant leg while standing and without contraction. Based on the manufacturers' recommendations, one of the following three sizes of the compression sleeve was used according to the calf and thigh circumferences:

- Size 1: 45–60 cm thigh +30–34 cm calf (*n* = 3 players)
- Size 2: 50–65 cm thigh +34–38 cm calf (*n* = 8 players)
- Size 3: 55–70 cm thigh +38–42 cm calf (*n* = 1 player)

The compression sleeves cover the lower extremity from the upper thigh to the phalanges. According to the manufacturer, the compression sleeves provide the following compression levels: ankle–25 mmHg, calf–19 mmHg, thigh–19 mmHg. However, the pressure levels of compression clothing were not measured in the participants.

### 2.7 Recovery period

During the first 90‐min recovery period, water, bananas, and muesli bars (CORNY classic; 452 kJ and 16.8 g carbohydrate per bar) were provided to players to be consumed ad libitum in their first experimental session, with consumption levels matched for the subsequent experimental session. The recovery period was spent in the two conditions in a randomized order: wearing compression sleeves (compression condition) or wearing no compression sleeves (control condition). The compression condition involved wearing commercially available full leg compression sleeves. The full leg compression sleeves were put on within the first 3 min of the recovery period. During the control condition, loose‐fit gymnastic pants without any compression were worn. Participants remained in a passive, seated position in the temperature‐controlled laboratory for the duration of the recovery period in both conditions. The order of the experimental conditions was randomized for each group, meaning that all members of a given group performed the same condition in the same order.

### 2.8 Outcome measures

#### 2.8.1 Repeated sprint performance

All 5 × 30 m sprint times were recorded in RSP_1_ and RSP_2_.

#### 2.8.2 BORG scale

The BORG Scale (6–20) is a retrospective evaluation to determine the subjectively perceived exhaustion during physical exercise (Borg, 1982). After each 15 min bout of the LIST protocol, participants were asked to rate their subjective perceived exhaustion.

#### 2.8.3 Heart rate measurements

In a subsample of the participants (*n* = 5), heart rates were recorded continuously with a heart rate monitor and chest strap (Polar H7, Polar Electro© Oy, Kempele, Finland) during the LIST protocol.

#### 2.8.4 Delayed onset of muscle soreness

A 10 cm visual analogue scale (VAS) was used to rate the subjective perception of exercise‐induced muscle soreness of the lower extremities 14 and 24 h post‐exercise for both conditions. Participants answered the question “How intense is your muscle soreness in your legs?” from “no soreness” (0 cm) to “maximal soreness” (10 cm). Quantification of perceived muscle soreness in the lower extremities was measured in millimeters, the distance between the left end of the scale (no soreness) to the participant's mark. The lowest possible score was 0, the highest was 100.

#### 2.8.5 Belief effect

Before the start of the measurements, participants were asked to answer the question “Do you believe that compression garments can improve your recovery process between two exercise bouts?” with a “yes” or “no” to evaluate a belief effect. According to the answer, subgroups were established to enable a subgroup analysis. Participants answering “yes” were allocated to subgroup “believers,” participants answering “no” were allocated to subgroup “non‐believers”.

### 2.9 Sample size calculation

An a priori sample size calculation was performed using G*Power (version 3.1). Based on the randomized crossover design, sample size was estimated for within‐subject comparisons of both repeated sprint performance and exercise‐induced muscle soreness (DOMS). For sprint performance, a moderate within‐subject effect size was assumed (Cohen's dz. = 0.80), with an alpha level of 0.05 and statistical power of 0.80, resulting in a required sample of 12 participants (Faul et al., 2007).

For DOMS outcomes, which typically demonstrate greater inter‐individual variability, a more conservative moderate effect size was assumed (Cohen's dz. = 0.65). Under the same statistical assumptions (*α* = 0.05, power = 0.80), the required sample size was 10 participants. To account for potential dropouts and to ensure adequate power for both performance and perceptual outcomes, 12 participants were recruited (Faul et al., 2007).

### 2.10 Statistical analysis

All data are reported as mean values (mean) and SDs. Total sprint times were extracted from the Fusion Sport online platform and collected in Office Excel 2016 (Microsoft Corporation, Washington, USA). The same procedure was performed for the heart rate data stored on the Polar Team App (Polar Electro, Kempele, Finland). The maximum heart rate percentages were based on the equation for the maximum heart rate = 220–age (Karvonen & Vuorimaa, 1988). Based on the sprint times assessed in the RSP_1_ and RSP_2_, the following variables were calculated: Sprint_best_ (= fastest sprint of the 5 sprints), Sprint_mean_ (= mean time of the 5 sprints), Sprint_drop_ (= Sprint_mean_ – Sprint_best_).

The software SPSS Statistics IBM, Version 25.0 (IBM, New York, USA) was used for further statistical analysis. Normal distribution was analyzed for each variable using the Kolmogorov–Smirnov test and variance homogeneity with the Levene Test.

To test for significant differences between the two conditions (compression condition vs. control condition) for sprint performance and post‐exercise DOMS, a two‐way repeated measures ANOVA was conducted with condition (compression condition vs. control condition) and time (RSP_1_ vs. RSP_2_ for sprint performance; 14 vs. 24 h post‐exercise for DOMS) as within‐subjects factors. To account for the increased risk of type I error due to multiple comparisons, *p*‐values were adjusted using the Benjamini‐Hochberg procedure. To assess the effect sizes of the independent variable (compression condition and the control condition) on the dependent variables, partial eta squared (*η* ^2^ₚ) was calculated for the ANOVA results (Shaffer, 1995). According to Cohen (1992) the following values of *η* ^2^ₚ represent small (*η* ^2^ₚ ≈ 0.02), medium (η^2^ₚ ≈ 0.15), and large (*η* ^2^ₚ ≈ 0.35) effects (Cohen, 1992). The statistical significance level was set a priori at *p* < 0.05 for all analyses before adjustment.

To test for significant differences in the two conditions (compression condition vs. control condition) for performance change (mean sprint time) from RSP_1_ to RSP_2_, student's *t*‐test for paired samples was performed. Also, the value between each condition was evaluated by calculating the effect size Cohen's d (difference between the means/pooled SD; Cohen's d, *d*) to estimate practical relevance, with *d* ≥ 0.2 indicating small, *d* ≥ 0.5 medium, and *d* ≥ 0.8 large effects (Lakens, 2013). Cohen's d effect sizes for unpaired samples were calculated since the Cohen's d effect size calculation for paired samples tends to yield very high effect sizes, which may lead to an overestimation of effect sizes (Shaffer, 1995).

## 3 RESULTS

### 3.1 Belief in compression

11 out of 12 soccer players (91.7%) believed that COMP may improve their recovery process. One (8.3%) out of the 12 players did not believe that COMP would improve his recovery process.

### 3.2 Heart rate during LIST

The mean heart rate during the three bouts of the modified LIST protocol for the subsample of five soccer players was 170 ± 4 bpm (83.6 ± 2% of the calculated HR_max_) for COMP and 165 ± 2 bpm (81.1 ± 1% of the calculated HR_max_) for CON. Details of HR during the three LIST bouts are displayed in Table 2.

**TABLE 2:** Heart rate (mean ± SD) in a subsample of five youth soccer players during the modified LIST protocol and perceived exertion levels (mean ± SD) in the 12 youth soccer players during the modified LIST protocol.

	Heart rate [bpm] during LIST		Ratings of perceived exertion after LIST	
	Compression condition	Control condition	Compression condition	Control condition
Total LIST	169.7 ± 11.9	164.5 ± 12.6	13.5 ± 1.7	13.3 ± 1.2
LIST‐bout 1	170.8 ± 10.5	167.2 ± 11.5	13.2 ± 2.1	13.3 ± 1.5
LIST‐bout 2	165.2 ± 13.3	163.2 ± 14.7	13.2 ± 1.8	13.3 ± 1.2
LIST‐bout 3	173.2 ± 14.2	163.2 ± 15.4	14.1 ± 1.1	13.3 ± 1.0

Abbreviations: Compression condition, wearing full leg compression sleeves during the recovery period; Control condition, wearing gym pants during the recovery period; HR, heart rate; LIST, modified Loughborough Intermittent Shuttle Test.

### 3.3 Perceived exertion during LIST

The perceived exertion levels during the modified LIST protocol for the 12 soccer players during the two different conditions are displayed in Table 2.

### 3.4 Repeated sprint performance

**FIGURE 2:**
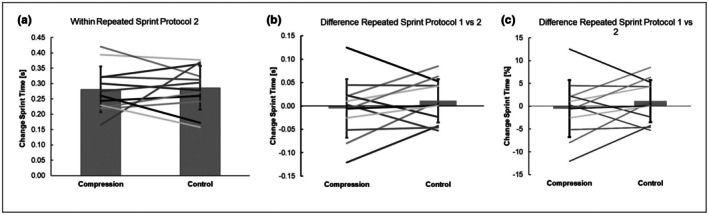
Individual performance drop (lines) of the 12 youth soccer players in both conditions (compression condition and control condition. (a) repeated sprint performance during RSP2; (b) performance change of mean sprint time (in seconds) from RSP1 to RSP2; (c) performance change of mean sprint time (in %) from RSP1 to RSP2. Bar graphs represent the mean values of 12 players. Compression, compression condition (wearing full leg compression sleeves during recovery period). Control, control condition (wearing no compression sleeves during recovery period).

The results for the sprint measurements for both conditions, compression condition and control condition, are displayed in Table 3, Figure 2. After adjusting for multiple comparisons using the Benjamini‐Hochberg procedure, there were no significant interactions and main effects of time or condition between condition (compression condition vs. control condition) and time (Sprint 1 vs. Sprint 2) for mean sprint time, best sprint time, or performance drop.

**TABLE 3:** (Mean ± SD) of repeated sprint performance in both RSPs in the 12 youth soccer players.

Parameter	Group	RSP_1_	RSP_2_	Group by time interaction, adjusted *p*‐value (partial eta squared)	Main effects, adjusted *p*‐value (partial eta squared)	
					Time	Condition
Mean sprint time [s]	Compression condition	4.59 ± 0.16	4.65 ± 0.18	0.790 (0.091)	0.962 (0.004)	0.255 (0.418)
	Control condition	4.59 ± 0.15	4.64 ± 0.19			
Best sprint time [s]	Compression condition	4.33 ± 0.15	4.37 ± 0.17	0.961 (0.002)	0.802 (0.137)	0.135 (0.410)
	Control condition	4.31 ± 0.16	4.35 ± 0.20			
Performance drop [s]	Compression condition	0.26 ± 0.08	0.29 ± 0.06	0.694 (0.041)	0.639 (0.058)	0.696 (0.088)
	Control condition	0.28 ± 0.07	0.29 ± 0.07			
Performance drop [%]	Compression condition	6.03 ± 1.77	6.54 ± 1.84	0.677 (0.035)	0.660 (0.079)	0.686 (0.062)
	Control condition	6.56 ± 1.31	6.60 ± 1.78			

Performance change (mean sprint time) from RSP_1_ to RSP_2_			*p*	Cohen's d
Performance change (mean sprint time) from RSP_1_ to RSP_2_ [s]	Compression condition	−0.01 ± 0.06	0.316	0.181
	Control condition	+0.01 ± 0.05		
Performance change (mean sprint time) from RSP_1_ to RSP_2_ [%]	Compression condition	+0.53 ± 6.24	0.316	0.300
	Control condition	−1.12 ± 4.63		

Abbreviations: Compression condition, wearing compression sleeves during the recovery period; Control condition, wearing gymnastic pants during the recovery period.

### 3.5 Delayed onset of muscle soreness (DOMS)

**FIGURE 3:**
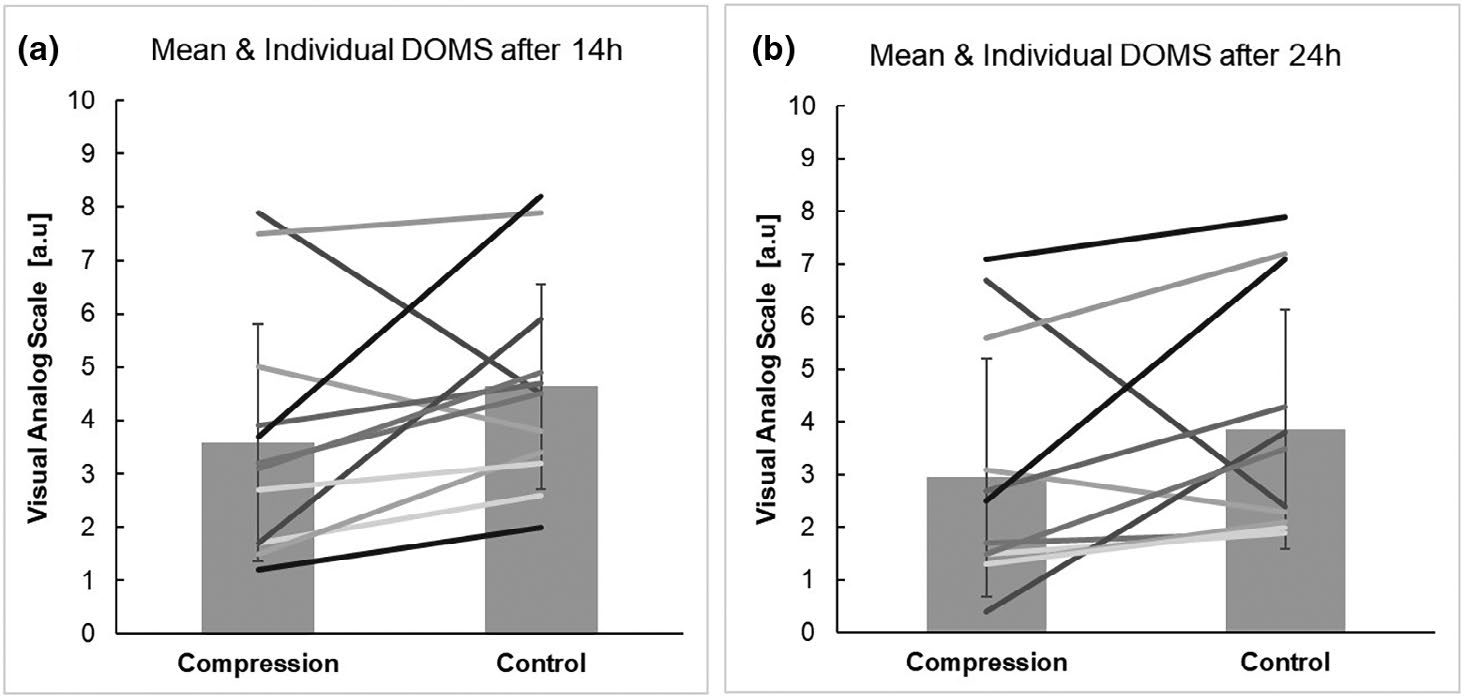
Individual ratings (lines) of delayed onset of muscle soreness in 12 youth soccer players in both compression condition and control condition. (a) DOMS 14 h following RSP_2_; (b) DOMS 24 h following RSP_2_. Bar graphs represent the mean values of 12 players. DOMS, delayed onset of muscle soreness; Compression, compression condition (wearing full leg compression sleeves during recovery period); Control, control condition (wearing no compression sleeves during recovery period).

The results of DOMS 14 and 24 h post‐exercise for both conditions, compression condition and control condition, are displayed in Table 4, Figure 3. After applying the Benjamini‐Hochberg adjustment, there were no significant interactions or main effects for the DOMS measurements.

**TABLE 4:** (Mean ± SD) of delayed onset of muscle soreness at 14 and 24 h post‐RSP_2_ in the compression and control conditions.

	Group	14 h following RSP_2_	24 h following RSP_2_	Group by time interaction, *p*‐value (partial eta squared)	Main effects, *p*‐value (partial eta squared)	
					Time	Condition
Delayed onset of muscle soreness (0–10)	Compression condition	3.49 ± 1.73	2.78 ± 2.32	0.912 (0.001)	0.831 (0.106)	0.240 (0.310)
	Control condition	4.73 ± 2.32	4.04 ± 2.12			

Abbreviations: Compression condition, wearing full leg compression sleeves during recovery period; Control condition, wearing no compression sleeves during recovery period.

## 4 DISCUSSION

The primary goal of the present study was to assess the effects of lower limb full‐leg compression sleeves, worn during a 90‐min passive recovery period after a soccer‐specific load, on repeated sprint performance and post‐exercise DOMS in well‐trained youth soccer players. The main findings of the present study were the following:

1. Repeated Sprint Performance: There was no significant difference in the change of mean sprint time from the first repeated sprint protocol to the second repeated sprint protocol between the compression and control condition. Specifically, the mean sprint times remained relatively stable in both conditions (compression condition: 4.59 ± 0.16 s to 4.65 ± 0.18 s; control condition: 4.59 ± 0.15 s to 4.64 ± 0.19 s). After adjusting for multiple comparisons using the Benjamini‐Hochberg procedure, the interaction between condition and time was not significant (adjusted *p* = 0.790, *η* ^2^ₚ = 0.091). There were also no significant main effects of time (adjusted *p* = 0.962, *η* ^2^ₚ = 0.004) or condition (adjusted *p* = 0.255, *η* ^2^ₚ = 0.418) on mean sprint time.
2. Delayed Onset of Muscle Soreness: Participants reported lower DOMS scores at both 14‐ and 24‐h post‐exercise in the compression condition compared to the control condition; however, these differences were not statistically significant after adjusting for multiple comparisons. At 14 h post‐exercise, DOMS scores were 3.49 ± 1.73 for the compression condition and 4.73 ± 2.32 for the control condition. At 24 h post‐exercise, scores were 2.78 ± 2.32 for the compression condition and 4.04 ± 2.12 for the control condition. The interaction between condition and time was not significant (*p* = 0.912, *η* ^2^ₚ = 0.001), nor were the main effects of time (adjusted *p* = 0.831, *η* ^2^ₚ = 0.106) or condition (adjusted *p* = 0.240, *η* ^2^ₚ = 0.310).

Wearing full‐leg compression sleeves during the 90‐min passive recovery period following a soccer‐specific load did not have a significant effect on repeated sprint performance, as the changes from RSP_1_ to RSP_2_ were not significantly different between the two conditions. The absence of performance change between RSP_1_ and RSP_2_ was consistent across both compression condition and control condition, indicating that this outcome was independent of compression sleeve use. Additionally, although participants reported lower DOMS in the compression condition compared to the control condition, these differences were not statistically significant after adjusting for multiple comparisons. This suggests that the observed trend toward reduced muscle soreness with compression sleeves may not be attributable to the intervention. These findings highlight that wearing compression sleeves may not provide a significant benefit in attenuating post‐exercise muscle soreness or enhancing recovery in youth soccer players. Therefore, the efficacy of compression garments on recovery remains inconclusive in this context, underscoring the need for cautious interpretation and further research to conclusively determine their effectiveness.

The present findings are not in line with previous studies examining the effect of compression clothing worn during a recovery period between two exercise bouts and reporting positive effects of compression clothing on exercise performance in the second exercise bout (Brophy‐Williams et al., 2017; de Glanville & Hamlin, 2012; Driller & Halson, 2013). However, these three studies assessed endurance performance in running (Brophy‐Williams et al., 2017) and cycling (de Glanville & Hamlin, 2012; Driller & Halson, 2013) with a considerably longer exercise duration in an adult population and are therefore not directly comparable with the settings and findings of the present study. In the study of Zinner et al. (2017) involving adult male handball players, the compression tights worn during the post‐exercise period following a repeated and exhausting sprint exercise did not improve subsequent repeated sprint and jump performance. Comparing our adolescent players with those in Zinner et al. (2017) study might be inappropriate due to differences in physiological development and training levels. However, the underlying physiological mechanisms of compression garments for recovery, such as improved blood flow and reduced muscle soreness, are relevant across age groups. Nevertheless, in the study of Zinner et al. (2017) the compression tights were worn for 48 h but in the present study we focused on the acute effects (i.e., after 90 min) of wearing compression clothing between the two repeated sprint efforts. The absence of ergogenic effects of compression clothing was also documented in previous studies (Duffield et al., 2010; Duffield & Portus, 2007; Ravier et al., 2018; Zinner et al., 2017). Studies demonstrating clear performance enhancing effects on repeated sprint performance while wearing compression garments during recovery are sparse.

So far one study demonstrated positive and practical relevant effects on repeated sprint performance when compression garments were applied during a recovery period of 24 h following a simulated rugby match (Hamlin et al., 2012). But the majority of studies with an initial simulated match or an initial intense bout of repeated sprinting and a subsequent recovery period with wearing compression garments followed by a repeated sprint protocol demonstrated no positive effects of compression clothing on (repeated) sprint performance (Atkins et al., 2020; Duffield et al., 2008; Duffield & Portus, 2007; Goto et al., 2017; Zinner et al., 2017).

One important observation of the present study was that after 90‐min of recovery the performances in RSP_2_ were very similar to the initial RSP_1_ performances in both conditions. Considering the absence of a profound change in repeated sprint performance from RSP_1_ to RSP_2_ in both conditions it might be suggested that the exercise demand of the modified LIST protocol, which was performed after RSP_1_ and before RSP_2_, was not sufficient to induce severe fatigue and/or exercise‐induced muscle damage to reveal the benefit of wearing compression sleeves when compared to the control condition during the recovery period. Indeed, the mean heart rate during the LIST corresponds to 83.6% of HR_max_ and a perceived exertion of 13.5 in the compression condition and to 81.1% of HR_max_ and a perceived exertion of 13.3 in the control condition reflected moderate to high intensity but not a maximum intensity (Pescatello, 2014).

The perceived post‐exercise muscle soreness in the lower extremities was not lower when wearing compression sleeves during the 90 min recovery period. The partial eta squared effect sizes of post‐exercise DOMS indicate medium effect sizes, suggesting a certain practical relevance. Indeed, the majority of comparable studies demonstrated a reduced post‐exercise DOMS in working muscles when compression clothing were applied during the recovery period between two exercise bouts (Brophy‐Williams et al., 2017; Goto et al., 2017; Lee et al., 2021) and/or for a more extended period during the post‐exercise phase (Davies et al., 2009; Hamlin et al., 2012; Upton et al., 2017) as well as when compression clothing were worn during exercise (Valle et al., 2013). Although, one should distinguish between studies in which compression garments were applied during rather short recovery periods of 60–90 min between two exercise bouts (Brophy‐Williams et al., 2017; Lee et al., 2021) and those studies applying compression clothing for longer post‐exercise periods of >12 h (Davies et al., 2009; Hamlin et al., 2012; Upton et al., 2017; Zinner et al., 2017).

The underlying mechanisms of reduced DOMS with compression clothing may be due to physiological and psychological mechanisms, but neither of these potential underlying mechanisms was measured and/or analyzed in the present study. Nevertheless, potential underlying mechanisms for reduced post‐exercise DOMS with compression garments could be attributable to reductions in swelling and oedema (Brown et al., 2022) and reductions in muscular inflammation (Brown et al., 2022; Mizuno et al., 2017; Valle et al., 2013). Only one study has examined the effect of compression garments on exercise‐induced intramuscular inflammation (Valle et al., 2013), and the results demonstrated that intramuscular inflammation of the working muscles was lower when wearing compression shorts (Valle et al., 2013). Since oedema seems to facilitate the inflammatory response of the working muscle (Lawrence & Springer, 1991), it may be beneficial to wear compression clothing during the post‐exercise period to maintain a proper hemodynamic flow (Liu et al., 2008), which bears the potential to reduce oedema and an inflammatory response.

Studies investigating the impact of compression garments on exercise performance have shown that the belief in an ergogenic effect linked to wearing compression clothing appears to enhance exercise performance (Brophy‐Williams et al., 2017). The belief effect refers to the individual perceived efficacy of the compression garment to aid recovery, meaning that individuals believing that compression clothes aid the recovery process actually exhibit a better exercise performance or a lesser decrease of exercise performance following a recovery period with compression clothing compared to individuals that do not believe compression clothes can aid the recovery process (Brophy‐Williams et al., 2017). The theory of a belief effect and the potential impacts on exercise performance have been discussed in detail previously (Halson & Martin, 2013; McClung & Collins, 2007). Although we assessed the perceived efficacy of the compression garment to aid the recovery process in all participants at the start of the study, we were not able to perform an analysis with respect to the belief effect since all but one participant believed that compression clothing can improve the recovery process. Even though 11 out of 12 players were believers, no positive effect of compression clothing on repeated sprint performance was evident. Indeed, the positive impact of compression sleeves diminishing the post‐exercise DOMS may be induced by belief effect. But this assumption remains speculative since the results of the present study did not allow us to perform a subgroup analysis with respect to believers vs. non‐believers. However, numerous studies (Brophy‐Williams et al., 2017; Goto et al., 2017; Lee et al., 2021; Valle et al., 2013) and reviews (Altarriba‐Bartes et al., 2020; Hill et al., 2014) demonstrated the positive effect of compression clothing on post‐exercise DOMS in various scenarios.

One limitation of this study is the small sample size, which may affect the generalizability and robustness of the findings. As limitations of the present study, we consider the absence of measuring the pressure levels of compression clothing in the participants. This might increase the possibility of varying pressure levels among the participants or insufficient levels of garment pressure, which might have masked the efficacy of compression sleeves. In addition, the lack of substantial changes in repeated sprint performance from RSP_1_ to RSP_2_ did not allow the wearing of compression sleeves during the recovery period to reveal any potential benefits. The absence of a performance change between the two repeated sprint protocols suggests that this outcome was independent of the condition. Therefore, future studies should apply more strenuous exercise bouts before the recovery period yielding greater performance changes. Lastly, the sample of 12 well‐trained youth soccer players in our study was rather small, thereby limiting the generalizability of our findings.

## 5 CONCLUSIONS

In the present study, wearing lower‐leg compression sleeves during a 90‐min recovery period following a soccer‐specific intensive load had no impact on repeated sprint performance and no effects on exercise‐induced leg soreness 14 and 24 h afterwards in a sample of well‐trained youth soccer players. Therefore, in this specific context, the application of full leg compression sleeves in a recovery period after soccer‐specific intense exercise in trained youth soccer players is not an effective tool to increase recovery or reduce post‐exercise muscle soreness. Future studies should examine the impact of compression sleeves following more intense soccer‐specific exercises, inducing a stronger performance change.

## AUTHOR CONTRIBUTIONS

Stefan Altmann, Rainer Neumann, and Florian A. Engel substantially contributed to the development and design of the study. Stefan Altmann, Rainer Neumann, and Florian A. Engel conducted the practical execution and data collection with the soccer players, both in the laboratory and in the field. Stefan Altmann and Florian A. Engel analyzed and interpreted the data. Claudia Kubica, Stefan Altmann, Billy Sperlich, and Florian A. Engel drafted the manuscript. All authors contributed to the manuscript's development, with Billy Sperlich and Stefan Altmann providing critical revisions. All authors read and approved the final version of the manuscript.

## FUNDING INFORMATION

The authors have nothing to report.

## CONFLICT OF INTEREST STATEMENT

The authors declare no conflicts of interest.

## Supporting information


**Data S1.** CONSORT 2010 checklist of information to include when reporting a randomized trial.

## Data Availability

The data supporting the findings of this study are available from the corresponding author upon reasonable request.
